# Professional Values of Undergraduate Students at a Nursing School in South Africa

**DOI:** 10.1155/2023/9635033

**Published:** 2023-09-04

**Authors:** Portia Bimray, Jennifer Chipps, Victoire Ticha

**Affiliations:** School of Nursing, Faculty of Community and Health Sciences, University of the Western Cape, 14 Blanckenberg Street, Bellville, South Africa

## Abstract

**Background:**

Nursing schools play an important role in instilling nursing professional values in undergraduate nursing students and ensuring that they produce professional nurse graduates. Several studies in various countries have been conducted to describe the professional values held by nursing students, but this has not been explored in detail in South Africa.

**Aim:**

The purpose of this study was to describe the professional values held by undergraduate degree students at a nursing school in South Africa.

**Methods:**

A cross-sectional survey using a self-administered questionnaire was conducted. With a population of 1,233 undergraduate nursing students across four years in the degree programme at the nursing school, a sample of 294 was calculated as the representative (95% CI, 5% error, and 50% response distribution). The 26-item nurses professional values scale revision (NPVS-R) with five value dimensions was used to collect the data. Means, frequencies, and confidence intervals were used to describe the values and Mann–Whitney *U* tests and Kruskal–Wallis independent sample tests were used to compare the findings with the demographic characteristics.

**Results:**

A total number of 245 respondents completed the questionnaire (response rate of 83.3%). Overall, the nurse professional value score was high (113.1 ± 13.1). The values of trust (4.46 ± 0.61), justice (4.39 ± 0.57), and caring (4.38 ± 0.55) were rated significantly higher than those of professionalism (4.23 ± 0.64) and activism (4.22 ± 0.57). First- and final-year students had significantly higher professional value scores.

**Conclusion:**

The study results describe the professional values of undergraduate nursing students in the school and confirmed the importance of trust, justice, and caring as the key professional values in the South African setting. *Clinical Relevance*. Nursing education should embed and monitor nursing professional values in the curriculum. Instilling nursing professional values in undergraduate nurses during formal training programmes improves quality patient care and service delivery for clinical practice.

## 1. Introduction

Professional nursing values are the foundation of daily nursing care practice [1] and a corner stone in the selection and training of future nurses [2, 3]. To ensure quality ethical nursing care [4, 5], these values serve as a guideline for their professional behavior [6]. These values reflect the integrity and professional identity of the nurse and reinforce performance in practice [6]. As a new nursing student, it is important for students to start the process of professional values on entry as there is a relationship between the level of nurses' education and professional values [3, 7]. This was also supported by Arries [8], Bijani et al. [2], and Green [9], who suggest that the level of maturity of, i.e., junior and senior students, plays a role in the formation of professional values and was recommended for consideration for curriculum development [10, 11]. For new nursing students, professional values are rooted in personal values derived from their own culture and society [6] and are influenced by many other factors such as education, attitude, religion, ethnicity, and culture [6, 12].

The responsibility of professional nursing programmes therefore is to embed and integrate professional values throughout the nursing curriculum and monitor the process of acquisition and practice of core values that occurs throughout the nurse's lifetime [7, 13–15]. The paradigm of nursing and nursing education emphasises the need for professional values such as trust and caring [12] and the importance of nurse graduates to be able to demonstrate these values in practice [9].

Embedding professional values in the curriculum requires the need to monitor professional values as an educational objective in nursing education. Research scholars have developed valid and reliable quantitative scales to measure nurses' professional values with the most utilized instrument being the nurses professional values scale-revised (NPVS-R), developed by Weis & Schank [16] and based on the 2001 American Nurses Association's Code of Ethics for Nurses. The aim of this cross-sectional study was to describe the values held by undergraduate learner nurses in a school of nursing in South Africa, by using the nurses professional values scale which was developed by Weis and Schank [16].

## 2. Participants and Methods

### 2.1. Aim

The purpose of this study was to describe professional values held by undergraduate degree students at a nursing school in South Africa.

### 2.2. Design

A cross-sectional survey using a self-administered questionnaire was conducted with undergraduate student nurses at a university in the Western Cape in South Africa.

### 2.3. Study Population and Sampling

The study population had 1,233 nursing students registered for the undergraduate nursing program at a school of nursing at a university in the Western Cape in South Africa at the time of the survey. Using a 95% confidence level, a 5% margin of error, and a 50% response distribution, a sample of 294 was calculated, and using quota sampling per year level, all students were asked to participate.

### 2.4. Data Collection

This study utilizes data from a larger data collection project on values-based leadership. Data were collected, with permissions from the university and the school of nursing, through face-to-face self-administered questionnaires from 29^th^ October 2019 to 5^th^ November 2019. Appointment schedules were drawn up to meet with year-level coordinators of six-year levels to arrange for an information session and a suitable timeslot in which the nature and purpose of the project were explained to the participants. They could then ask clarifying questions about the project. Written permission was obtained from participants who chose to voluntary participate in the project. Academic staff administered the questionnaire after class sessions.

The inclusion criteria were that all students had to be registered in the legacy four- or five-year degree program. Only 245 participants completed the questionnaires. Forty-nine students of the calculated sample of 294 voluntarily decided not to partake in the study.

### 2.5. Instrument

The questionnaire included the validated nursing professional values scale (NPVS-R-5) (Weis & Schank, 2009). The NPVS-R has previously been used in South Africa and comprises 26 items representing five professional value dimensions, namely, justice (five statements: 1, 2, 9, 14, and 15), trust (statements: 3, 12, and 13), professionalism (four statements: 5, 6, 7, and 8), caring (nine statements: 16, 17, 18, 20, 21, 22, 23, 24, and 25), and activism (five statements: 4, 10, 11, 19, and 26). The NPV-R scale used a 5-point Likert scale rating the importance of each individual statement with 1 rated as not important, 2 somewhat important, 3 important, 4 very important, and 5 most important. The overall professional value score is out of 130 with a range of 26–130. More importance an individual ascribes to a value statement was reflected in a higher total score, with scores below 43, between 43 and 86, and above 86 classified as overall low, medium or moderate, and high importance of professional values, respectively [7]. Studies conducted in Nigeria, Iran, Israel, Spain, and Turkey demonstrated scale reliability of the scale with a range of Cronbach Alphas from *α* = 0.81 to *α* = 0.96 [1, 3, 7, 9, 13]. This was also confirmed in this study (*α* = 0.941). To further ensure reliability, a pretest study was conducted with 10 student nurses in their third year of study. No changes were made to the tool following the pretest, and the responses were included in the results.

### 2.6. Data Analysis

Data were analyzed using SPSS version 28. Demographics and scale dimensions were reported using descriptive statistics (frequency, percentage, mean, 95% confidence intervals, and standard deviation) and Mann–Whitney *U* and Kruskal–Wallis independent sample tests to compare scores across demographics. The level of significance was set at *p* < 0.05.

## 3. Results

A total number of 245 respondents completed the questionnaire (a response rate of 83.3%). The respondents had an average age of 22.2 (±3.6) years, and most of the respondents were female (210, 85.7%). The respondents were evenly distributed across year levels with 62 (25.3%) in year 1, 62 (25.3%) in year 2, 62 (25.3%) in year 3, and 58 (23.7%) in year 4 (Table 1). Respondents reported having been in the school on average for 2.7 ±1.3 years, ranging from 1 to 7 years.

**Figure 1:**
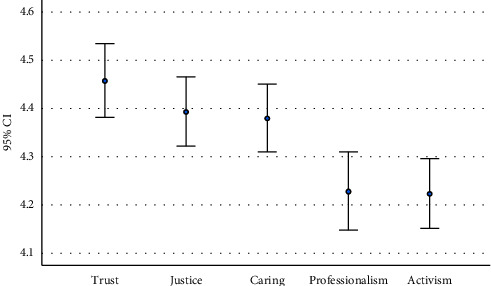
Professional values.

Overall, the importance of professional values from the respondents' perspectives was high (113.1 ± 13.1), with no significant differences between genders (*p*=0.102, Table 1). Nearly, all respondents rated professional values as very important (197 (80.4%)) and only 11 (4.5%) as medium important. There were significant differences between year levels with year 1 and year 4 respondents (115.9 and 114.2, respectively) reporting significantly higher levels of importance of professional values than years 2 and 3 (112.8 and 109.6, respectively, *K* = 11.1, *p*=0.025) (Table 1). In terms of the professional values, trust (4.46 ± 0.61), justice (4.39 ± 0.57), and caring (4.38 ± 0.55) were rated significantly higher than professionalism (4.23 ± 0.64) and activism (4.22 ± 0.57) (Figure 1).

The most important values from the respondents' perspective were “Maintaining confidentiality of patients” (4.59 ± 0.71), “Safeguarding patients' right to privacy” (4.58 ± 0.68), and “Protect moral and legal rights of patients” (4.53 ± 0.69) from the caring dimension, “Promote equitable access to health care” (4.53 ± 0.71) from the trust dimension, and “Maintain competency in area of practice” (4.53 ± 0.74) from the justice dimension (Table 2). The least important rated values were “Participate in peer review” (3.96 ± 0.90) from the professional dimension, “Participate in public policy decisions affecting distribution of resources” (3.88 ± 0.92) from the activism dimension, and “Refuse to participate in care if in ethical opposition to own professional values” (3.82 ± 1.16) from the caring dimension.

## 4. Discussion

This current study has shown high ratings of the importance of professional values by the nursing student respondents (113.1 ± 13.1) in this setting, similar to other studies that reported high scores for nursing students, as well as nurses' (who were at different levels of education and generations) professional values, using the NPVS-R [7–9]. This suggests that according to this scale, undergraduate nursing students in this school have achieved success in embedding professional values in nursing during the nurse training programme. This study also confirms another older South African study (Weis & Schank, 2009) who found high overall levels of professional values.

The most important value was trust, with the statement “Promote equitable access to health care,” ranked third amongst all the value statements, with a mean score of 4.53 (0.71). A reason for this high score could be attributed to the context of the study where there are still disparities between socioeconomic groups in South Africa [17], and many respondents come from low socioeconomic communities with poor access to health care. Access to quality services in South Africa has remained low despite many efforts and approaches by African countries to equalise health care [18].

Within the value dimension of caring, the highest ranked ratings in the scale were for the following: “Maintain confidentiality of patients,” “Safeguard patients” right to privacy,” and “Protect moral and legal rights of patients” which was rated 4^th^ highest. These findings were congruent with the findings of a study conducted in Iran which compared the professional values of nursing students, nurses, and nursing educators and found overall mean scores for all within the range of relatively important or important. Similarly, from the student nurse's perspective in Iran, statements which relate to caring such as “Maintain confidentiality of patients” and “Protect moral and legal rights of patients” had the highest importance ratings for nursing students [2].

Similar to other studies by Chikeme [3] and Poorchangizi et al. [7], the following statements were rated as less important in the current study: “Recognizing role of professional nursing associations in shaping healthcare policy” (ranked 20^th^), “Engaging in ongoing self-evaluation” (ranked 21^st^), “Participating in nursing research/implementing research findings appropriate to practice” (ranked 22^nd^), and “Participating in peer review” (ranked 24^th^). Though similar, the respondents in the Poorchangizi et al. [12] study ranked “Recognizing role of professional nursing associations in shaping healthcare policy” 24^th^, “Engaging in ongoing self-evaluation” 17^th^, “Participating in nursing research/implementing research findings appropriate to practice” 23^rd^, and “Participating in peer review” 25^th^, which could be attributed to certain factors in their settings such as educational semester, low motivation, insufficient affirmation, and low encouragement by nursing educators. Although students in this study are being taught about the importance of participating in peer review during their course of training for quality assurance purposes, no actual opportunities exist in undergraduate progammes where students can actively engage and involve themselves in peer-review activities. However, nurse educators do ensure the presence of the “student voice” during accreditation processes by the regulatory body of South Africa and opportunities where there are formal disciplinary hearings and curriculum reform at the institution of higher learning. However, not all students get the opportunity to be selected when these opportunities arise, and therefore, the majority of students might regard this item “Participating in peer review” as least important amongst the values because of not knowing what it actually entails.

A major concern was the low ranking (26^th^) of importance of the statement “Refusing to participate in care if in ethical opposition to own professional values,” in contrast with Poorchangizi et al. [12], whose respondents ranked this statement more important at 13^th^ amongst the value statements. This statement is from the caring value dimension and is central to ethical decision-making in practice [7]. Our finding may relate to the diverse context in South Africa with most of the student population having varied value orientations, but this would have to be investigated further. Another reason for the low ranking of this value statement could also be due to students not being exposed enough to real-life encounters of an ethical nature in practice, but this also requires more research.

A further concern is the low ranking of the statement “Participating in public policy decisions affecting distribution of resources” which ranked 25^th^ in this study and 26^th^ in the study by Poorchangizi et al. [12]. Health and public policy are essential elements of professional obligations of nurses [19]. Also, the American Nurses Association's [20] nursing Code of Ethics state that nurses have a responsibility to participate in political processes and advocate for their patients [21]. Though respondents rated “Acting as a patient advocate” as an important value under the caring dimension at 11^th^, “Participating in public policy decisions affecting distribution of resources” was ranked 25^th^ which is considered as the least important, highlighting a difference of a belief and translating this value into practice amongst the respondents. These values are taught in the nursing school, but being a student may provide limited ability to engage in health and public policy activities [22]. In addition, the current programme may not adequately equip students with the analytic, communication, and leadership skills to translate this into practice, but this should be further investigated [12]. This could be the same reason why students in this study scored the statement low, as not enough emphasis is placed on active involvement and engagement in public policy at student level. Also, in the current study, opportunities at policy discussion level are almost nonexistent in the programme and should be further explored by nurse educators to give students more exposure to boost their leadership skills.

There was no significant difference found in this study between professional values and gender (*p*=0.102). Nearly, all respondents rated professional values as very important (197 (80.4%)) and only 11 (4.5%) as medium important. The results of the current study were similar to those of a study that was conducted in Pakistan in 2018, which showed that male and female respondents paid equal attention to professional values [5]. However, the study confirmed previous papers which suggested that the level of education influenced the integration of values [10, 11, 13], with differences due to demographics related to academic levels of study. In all four-year levels, the respondents had high overall scores for professional values, but first- and final or fourth-year level students recorded significantly higher ratings in professional values than students in their second and third years (112.8 and 109.6, respectively, *K* = 11.1, *p*=0.025), though they might not consider each value and its corresponding statements as equally important [23]. The findings were in line with a study in Turkey [24] which indicated that the academic year had an effect on the professional values of nursing students, and [9] a study in Israel with novice (first year), advanced (third year), and senior (fourth year) students also found statistically significant differences between these groups. The high ratings in the first year in the current study were consistent with findings by Bleda et al. [13], who found that nursing students rated professional values as highly important when they enter the nursing profession because of some students having inadequate clinical experience to accurately rank the statements. In our study, the high scores of first-year respondents could relate to high levels of preexisting values which then further translate into professional values towards the end of their training [13]. Another reason could also be that South African nursing students come from different cultural backgrounds and enter the university with a set of diverse values that could influence how each student assigned meaning to the importance of professional values and how they scored the statements. The final-year respondents are also more mature students, preparing for practice as new graduates, which may reflect the preparation for practice [13]. Furthermore, the final-year respondents in this current study also have a formal professional practice module in their final year of study which exposes them to further growth and development of these values as they take on more responsibilities as senior students. This professional maturity is linked to chronological maturity as these students have been exposed to the need to make independent judgments in their professional and personal lives [8, 9]. Bijani et al. [2] also found a similar pattern with higher academic levels being associated with higher scores for professional values.

### 4.1. Limitations

There are some limitations in this study. First, respondents were from one university in the Western Cape Province of South Africa only, and though it confirmed 2009 South Africa study's findings, caution should be taken in generalizing these findings to undergraduate nursing students in South Africa. Furthermore, because of the diverse nature of the study population, religion, cultural background, language, and ethnic origin may influence the development of professional values but are not explored in terms of their association with nurses' professional values in this study. The study did not explore the reasons for the ratings, and further studies are needed to explore the factors that influenced the low ratings for professionalism and activism values.

## 5. Conclusion

Professional values in nursing students are central to their training as these values would determine quality patient care service delivery and guide new graduates in their ethical decision-making while in nursing practice. This study showed that the respondents rated professional values highly and that this may be due to the selection process of students entering the programme and the process of embedding professional values in the nursing curriculum.

## Figures and Tables

**Table 1:** Demographics and NPV-R scores (*n* = 245).

	*n* (%)	Mean (sd)	Test	*p* value
Gender (*n* = 245)				
Male	35 (14.3)	109.4 (14.6)	*U* = 1.6	0.102
Female	210 (85.7)	113.7 (12.8)		

Year level (*n* = 244)				
Year 1	62 (25.3)	115.9 (10.1)	*K* = 11.1	0.025
Year 2	62 (25.3)	112.8 (14.3)		
Year 3	62 (25.3)	109.6 (11.8)		
Year 4	58 (23.7)	114.2 (15.0)		

**Table 2:** Value dimensions and statements.

Value dimensions and statements	Rank	Mean (sd)
*Activism*		
Participate in activities of professional nursing associations	13	4.4 (0.77)
Advance the profession through active involvement in health-related activities	16	4.36 (0.74)
Recognize role of professional nursing associations in shaping healthcare policy	20	4.3 (0.76)
Participate in nursing research/implement research findings appropriate to practice	22	4.18 (0.82)
Participate in public policy decisions affecting distribution of resources	25	3.88 (0.92)

*Caring*		
Maintain confidentiality of patient	1	4.59 (0.71)
Safeguard patients' right to privacy	2	4.58 (0.68)
Protect moral and legal rights of patients	4	4.53 (0.69)
Provide care without prejudice to patients of varying lifestyle	6	4.48 (0.77)
Practice guided by principles of fidelity and respect for person	7	4.45 (0.73)
Act as a patient advocate	11	4.41 (0.76)
Protect rights of participants in research	14	4.4 (0.81)
Confront practitioners with questionable or inappropriate practice	23	4.17 (0.91)
Refuse to participate in care if in ethical opposition to own professional values	26	3.82 (1.16)

*Justice*		
Maintain competency in area of practice	5	4.53 (0.74)
Accept responsibility and accountability for own practice	8	4.45 (0.77)
Seek additional education to update knowledge and skills	9	4.43 (0.76)
Request consultation/collaboration when unable to meet patients' needs	15	4.36 (0.8)
Engage in ongoing self-evaluation	21	4.21 (0.8)

*Professionalism*		
Promote and maintain standards where planned learning activities for students take place	17	4.33 (0.74)
Establish standards as a guide for nursing practice	18	4.33 (0.77)
Initiate actions to improve environments of practice	19	4.32 (0.78)
Participate in peer review	24	3.96 (0.9)

*Trust*		
Promote equitable access to health care	3	4.53 (0.71)
Assume responsibility for meeting health needs	10	4.42 (0.75)
Protect health and safety of public	12	4.41 (0.8)

## Data Availability

The data that were used and analyzed to support the findings of this study are available from the corresponding author on request.

## Additional Points

*Clinical Resources*. (1) An instrument to measure professional nursing values: https://sigmapubs.onlinelibrary.wiley.com/doi/abs/10.1111/j.1547-5069.2000.00201.x. (2) Importance of professional values in nursing and healthcare: https://www.heraldopenaccess.us/openaccess/importance-of-professional-values-in-nursing-and-healthcare. (3) Professional nursing values: a concept analysis: https://onlinelibrary.wiley.com/doi/abs/10.1111/nuf.12211.
